# Enhancing opportunistic recruitment and retention in primary care trials: lessons learned from a qualitative study embedded in the Cranberry for Urinary Tract Infection (CUTI) feasibility trial

**DOI:** 10.1186/s12875-022-01796-7

**Published:** 2022-07-26

**Authors:** Oghenekome A. Gbinigie, Anne-Marie Boylan, Christopher C. Butler, Carl J. Heneghan, Sarah Tonkin-Crine

**Affiliations:** 1 Nuffield Department of Primary Care Health Sciences, University of Oxford, Oxford, UK; 2 NIHR Health Protection Research Unit in Healthcare Associated Infections and Antimicrobial Resistance, University of Oxford, Oxford, UK

**Keywords:** Primary health care, Family practice, General practice, Urinary tract infection, Qualitative research

## Abstract

**Background:**

Opportunistic recruitment in primary care is challenging due to the inherent unpredictability of incident conditions, and workload and time pressures. Many clinical trials do not recruit to target, leading to equivocal answers to research questions. Learning from the experiences of patients and recruiters to trials of incident conditions has the potential to improve recruitment and retention to future trials, thereby enhancing the quality and impact of research findings. The aim of this research was to learn from the trial experiences of UTI patients and recruiters to the Cranberry for UTI (CUTI) trial, to help plan an adequately powered trial of similar design.

**Methods:**

One-to-one semi-structured interviews were embedded within the CUTI feasibility trial, an open-label, randomised feasibility trial of cranberry extract for symptoms of acute, uncomplicated Urinary Tract Infection (UTI) in primary care. Interviews were conducted with a sample of: CUTI trial participants; non-CUTI trial UTI patients; and, recruiters to the CUTI trial. Verbatim transcripts were analysed thematically.

**Results:**

Twenty-six patients with UTI and eight recruiters (nurses and GPs) to the CUTI trial were interviewed. Three themes were developed around: reasons for participating in research; barriers to opportunistic recruitment; and, UTI patients’ experiences of trial procedures. Recruiters found that targeted electronic prompts directed at healthcare practitioners based in clinics where patients with incident conditions were likely to present (e.g. minor illness clinic) were more effective than generic prompts (e.g. desk prompts) at filtering patients from their usual clinical pathway to research clinics. Using a script to explain the delayed antibiotic trial group to patients was found to be helpful, and may have served to boost recruitment. For UTI patients, using an electronic diary to rate their symptoms was considered an acceptable medium, and often preferable to using a paper diary or mobile phone application.

**Conclusions:**

The use of targeted prompts directed at clinicians, a script to explain trial groups that may be deemed less desirable, and an appropriate diary format for patient-reported outcomes, may help to improve trial recruitment and retention.

**Supplementary Information:**

The online version contains supplementary material available at 10.1186/s12875-022-01796-7.

## Background

Recruitment to Randomised Clinical Trials (RCTs) can be challenging; Up to two-thirds of clinical trials do not achieve their target sample size [1, 2]. Under-powered trials lead to research wastage

In the UK, patients can be recruited to primary care trials through research-active GP surgeries. Typically, patients are recruited by General Practitioners and nurses based in the GP surgery who are trained in research activities [3]. Recruitment can take place in a dedicated research appointments and/or, particularly for trials of incident conditions, in the course of a routine clinic. Opportunistic recruitment is particularly challenging in General Practice, given the high workload and time-pressured consultations [4]. However, this recruitment approach is unavoidable for trials of incident conditions, such as acute Urinary Tract Infection (UTI). Understanding barriers and facilitators to trial recruitment, such as time pressures and potential lack of equipoise [5], is critical to tailoring processes to facilitate recruitment. Whilst some qualitative research has sought to address these challenges [3, 6], only a few studies have done so through the lens of primary care recruiters [7].

In addition to successfully recruiting patients, participant retention and data completeness help to ensure that research generates meaningful results, reducing attrition bias [8]. This can be particularly challenging when outcome reporting is participant dependent, and participants are asked to do something above and beyond taking a study treatment (such as completing a daily symptom diary).

The Cranberry for Urinary Tract Infection (CUTI) trial was a feasibility, randomised trial of cranberry extract for symptoms of acute, uncomplicated UTI [9]. Interviews with trial recruiters and UTI patients (within and outwith the trial) were embedded in the CUTI trial. One aim of these interviews was to learn from the trial experiences of UTI patients and recruiters, using this learning to help plan an efficacy trial of a similar design.

We therefore present the findings and lessons learned from interviews with recruiters and UTI patients, many of which have broader applicability to trials of other incident conditions in primary care.

## Methods

### Context and Recruitment

This interpretivist qualitative study involved interviews embedded within the CUTI trial, an open-label, randomised feasibility trial [10]. CUTI trial methods have been published elsewhere [11]. In brief, acute UTI patients were recruited from four General Practices in Oxfordshire, UK, and were randomly assigned to:

- 1) Immediate antibiotics.
- 2) Immediate antibiotics and immediate cranberry capsules,
- 3) Immediate cranberry capsules and back-up antibiotics to be used if symptoms worsened or did not improve within 3–5 days.

Semi-structured interviews [12] were conducted by the Chief Investigator (CI) of the CUTI trial (OAG, a female General Practitioner and DPhil student with formal training in qualitative research) with a sample of: CUTI trial participants; non-CUTI trial patients who had experienced a recent UTI; and, trial recruiters. CUTI trial participants were invited by telephone to be interviewed two weeks after enrolment to the trial, which coincided with the end of the daily electronic diary that they were asked to complete as part of the trial. Once the trial had closed to recruitment, all recruiters (thirteen in total) were invited to take part in the interview study. Electronic invitations, including an invitation letter and a participant information leaflet (PIL), were sent to recruiters. Recruiters opting for a telephone interview were posted an informed consent form and were asked to sign and date it, before posting it back to OAG. Fully informed, written consent was obtained from each participant prior to being interviewed.

### Participants

We aimed to conduct interviews with UTI patients (CUTI trial participants and non-CUTI trial UTI patients) and recruiters to the CUTI trial, with the final number determined by data saturation [13], that is, reaching a stage at which any data from later interviews would not meaningfully change the developing analysis [14]. By the end of the twenty-fourth UTI patient interview, data saturation was indicated [14]; no new concepts from the data were identified that changed the analysis. This was confirmed in the next three interviews that were conducted (interviews 25 to 27). However, there was a degree of convenience to the samples, in that there was a limited sample of people to select from (especially CUTI trial recruiters).

Non-CUTI trial UTI patients were recruited from a General Practice in Oxfordshire outwith the CUTI trial, in an area with more ethnic diversity compared with the CUTI trial practices (of note, CUTI trial practices all had experience of recruiting to RCTs). We employed a purposive maximum variation sampling strategy [15] with regard to: the recruiters’ site; the recruiters’ roles (nurse or GP); UTI patients’ age; and, UTI patients’ trial group.

### Data collection

A semi-structured interview guide [12] was used to explore participants’ experiences (see additional files 1 and 2). UTI patients were invited to discuss: their most recent UTI episode; help-seeking behaviour; self-care strategies; thoughts on non-antibiotic treatments; and, thoughts on and experience of taking part in the CUTI trial. CUTI trial recruiters were asked to share their initial thoughts on the study, their experience of recruitment to the trial, and their views on recruiting to a main trial of similar design. All interviews took place at a location of the participant’s choosing (e.g. their own home, GP surgery, or the Oxford Primary Care Health Sciences Department), were audio-recorded and professionally transcribed verbatim.

### Data analysis

Thematic analysis was used to analyse the data [16], with data collection and analysis taking place concurrently. Transcripts were read and audio-recordings were listened to several times for familiarisation by OAG, allowing immersion in the data. NVivo 12 software was used to organise the data and facilitate coding. First, codes relating to similar phenomena were grouped into categories, and subsequently themes and sub-themes were developed to describe the data. This was an iterative process conducted by OAG in discussion with AMB and STC. Once the thematic structure was finalised, theme labels were refined to describe the data within, with supporting quotes selected to illustrate the themes and sub-themes.

### Patient and Public Involvement

Four PPI contributors were involved with the CUTI feasibility trial and interview studies from the outset. They scrutinised all public facing documents (e.g. participant information leaflets) and interview topic guides, and informed dissemination plans.

## Results

Thirty-five interviews were conducted between August 2019 and March 2020 with: CUTI trial participants (*n* = 14, T1-T14); non-trial UTI patients (*n* = 13, NT1–NT13); and, recruiters (*n* = 8, R1-R8). One non-trial UTI patient interview (NT1) was withdrawn as she met an exclusion criterion (immunosuppressed). UTI patient interviews lasted 28—72 min (mean 54 min), and recruiter interviews lasted 20 to 33 min (mean 26 min). Participant characteristics are described in Table 1.

**Table 1:** Characteristics of interview participants

	**CUTI trial group 1 participants**	**CUTI trial group 2 participants**	**CUTI trial group 3 participants**	**Non-trial UTI patients**	**Nurse Recruiters**	**GP Recruiters**
Number	6	5	3	12	6	2
ID numbers	T2, T7, T10, T11, T12, T13	T4, T5, T6, T8, T14	T1, T3, T9	NT2-NT13	R1-R6	R7, R8
Mean age (years)	67	69	44	45	-	-
Age range	32–81	60–77	23–57	18–76	-	-
Ethnic Group	White, English—6	White, English – 3 White, British – 1 White, Welsh—1	White, English – 2 White, British – 1	White, English – 2 White, British – 4 White, Spanish – 2 White, Lithuanian – 1 White, Bulgarian – 1 White, European – 1 White, Australian—1	-	-
Marital Status	Married – 3 Widowed – 2 Divorced—1	Married – 4 Widowed—1	Single, never married—1 Separated—1 Married – 1	Single, never married – 5 Domestic Partnership – 2 Married – 4 Widowed—1	-	-
Employment Status	Retired – 4 Employed for wages – 1 Self-employed—1	Retired – 4 Self-employed—1	Employed for wages – 1 Out of work and looking for work – 1 Self-employed—1	Retired – 3 Employed for wages – 7 Unable to work, medically – 1 Student—1	-	-
Highest level of school/degree	GCSE level – 1 A-level – 1 College qualification or equivalent – 2 Vocational training—1 University Bachelor’s degree – 1	GCSE level – 2 College qualification or equivalent – 2 University Bachelor’s degree – 1	A-level – 1 College qualification or equivalent – 1 Vocational training—1	GCSE level – 1 A-level – 1 College qualification or equivalent – 1 University Bachelor’s degree – 4 Master’s degree – 3 Doctorate degree—2	-	-

Themes pertaining to UTI patients’ experiences of non-antibiotic treatments and delayed antibiotic prescribing have been published elsewhere [17]. In the present manuscript, we discuss the following three themes: 1) Reasons for participating in the CUTI trial and interview study; 2) Overcoming barriers to opportunistic recruitment; and 3) UTI patients’ experiences of CUTI trial procedures.

Additional Table 1 highlights key learning points from the three themes for recruiting to acute UTI trials in primary care, many of which have wider applicability to other trials of incident conditions. We discuss some of these learning points in more detail below.

### Theme 1 – Reasons for taking part in the CUTI trial and interview study

#### UTI patients’ perspectives

The potential to improve UTI treatment options played an important part in women’s reasons for taking part in the CUTI trial. They perceived taking part as a way of advancing research in this field, and felt that exploring non-antibiotic treatments for UTIs, like cranberry, was “worthwhile.” Women also spoke of personal benefits from taking part in research – such as avoiding antibiotics.

*I think it’s just the fact that you’re helping, you know, if you can get away from antibiotics all well and good*. *(Trial participant (T) 4).*

When asked, UTI patients commonly cited a lack of time as a reason that they perceived people might not wish to participate in research. This was especially the case with taking part in an interview study; interviews took roughly an hour, whereas daily electronic diary entries (completed as part of the CUTI trial) took approximately five minutes.

Women talked of being interviewed as part of a research study as something of an unknown entity, and consequently considered that some people might find the idea anxiety-provoking. Furthermore, the word ‘interview’ had negative connotations for some people.

*They might feel nervous about it… people associate interviews with nerves and fear…(T12)*

A suggestion was made for a different name to be used for the interview process, such as a “discussion,” to make the process sound less daunting.

#### The potential impact of incorporating placebo

Incorporating a placebo into a future trial was discussed with UTI patients. Whilst they understood the rationale for including a placebo, the prospect of being randomly assigned to receive immediate placebo gave many women pause for thought. They often recognised that placebo designs were an important part of scientific study. However, the notion that they might be assigned to receive no active treatment initially tipped the balance of agreeing to a delayed antibiotic approach in a negative direction. For some, it signalled a trivialisation of their illness.

*That’s a mental thing that you’re thinking, they don’t care…at least with the cranberry you think, ‘Well it might work, you know, it might be okay.’…you got this urinary tract infection and they’re just giving me a sugar pill…I want something that’s going to make me feel really better.’ …with sugar pills…it’s a no–no for me (T4).*

Some women called into question the use of an immediate placebo as the only treatment in the context of managing an infection, as compared with a non-infective pain syndrome. Whilst they were aware of the placebo effect on pain symptoms, they perceived that it would not be possible for a placebo to treat a genuine infection.

*If you’ve got an infection and you think you’re getting something to make it better, I can’t see how that will make it better… I really don’t believe that mind of matter could clear up an infection…(T6).*

For those who would be willing to engage, receiving a delayed antibiotic prescription in addition to a placebo was typically a deal-breaker.

*Knowing that I’ve got the delayed antibiotics I knew at any point I could start taking them… I would have felt in control enough to have been happy to have taken the placebo*.(T3)

*If it has placebo and delayed antibiotics, that’s, I would go for it but if it doesn’t have antibiotics, I wouldn’t go for it because it has no treatment*. (NT7)

### Recruiters’ perspectives

Recruiters’ decision to recruit to the trial was dependent on whether: they felt the research would help to answer an important question; they perceived that the research would be acceptable to patients; and, the research seemed achievable – that is, research procedures did not appear onerous.

*I thought it was achievable. I thought that patients would like it. I didn’t think it would be particularly difficult to recruit to… (R3—nurse recruiter)*

### Theme 2 – Overcoming barriers to opportunistic recruitment

Research clinicians manually typing CUTI trial reminder messages at the top of the electronic patient list for the minor illness clinic/on-call (duty) doctor electronic lists was perceived as a valuable way to encourage clinical staff to divert appropriate patients to the research clinic. In addition, sending direct, on-the-day, electronic screen messages to clinicians working in these clinics was similarly perceived to be useful. Whilst these messages were not automated and therefore more manually intensive than Egton Medical Information System (EMIS) prompts (on-screen prompts appearing in response to trigger words typed into the electronic patient record, suggesting that a patient may be eligible for the trial), these targeted alerts were perceived to be effective.

*What I found quite useful was…we have like a booking system for urgent on the day appointments, I’d kind of put a reminder on that if anyone had symptoms of UTI please send to X for research kind of thing, that seemed to work better than the desk prompts… (R5—nurse recruiter).*

Recruiters to the trial stated that a common reason for women declining trial participation was because they did not wish to be assigned to the delayed antibiotic group; this was confirmed by the trial screening and enrolment log data [9].

*I explained to her over the phone what the study was about but I think she was the one that said, “Oh no, I don’t want to delay treatment,” (R1—nurse recruiter)*

*They didn’t want to have the delayed antibiotic because they had a reason, i.e., they were convinced they had a UTI and [um] they had an exam the next day or there was something going on in their life which and they didn’t want to risk kind of feeling the way they were feeling for any longer than necessary if they got the delayed antibiotic (R6 – nurse recruiter).*

There was a suggestion from recruiters that there was a degree of conditioning of patient behaviour; there was an expectation from patients to receive an immediate antibiotic prescription, as this is what usually happens when women with UTIs present to healthcare practitioners.

*Some people come in with an agenda. A lot of people come in and, you know, plonk themselves down and say, “I’ve got a urinary tract infection. I need antibiotics,”* (R6).

*I think it was just that there’s almost, there’s that programmed, ‘an antibiotic will sort this out.’ (R2—nurse recruiter)*

The trial team created a script for recruiters to explain the delayed antibiotic group, framing this group as a way to potentially avoid taking antibiotics (see additional file 3). Recruiters generally found the script helpful; not only did they find that it helped them to clearly explain the delayed antibiotic group, but also that it helped recruitment.

*You can get a little bit tongue tied sometimes when you’re explaining something, when you’ve just got that [script], you know, with very, with clarity as to, to what is being said…that was useful…(R4 – nurse recruiter).*

*Having that [the script for the delayed antibiotics group] at the very beginning to have said would have been I think probably, probably missed a couple at the very beginning because they just said, “I want to take antibiotics. (R3—nurse recruiter).*

The trial team created desk prompts to be placed on the desks of clinical staff at recruiting sites (e.g. General Practitioners and nurses). Whilst some recruiters perceived that their clinical colleagues saw them as helpful, real-time reminders, they did not necessarily perceive that this translated into a meaningful diversion of eligible patients to research staff. There was potential for desk prompts to be ignored, exacerbated by the desk prompts being perceived as insufficiently “eye-catching.”

*I don’t know whether people just kind of they have it there but tend to ignore it a little bit. (R5—nurse recruiter*).

*They were a bit small and blended into the background a bit I think a bit too much. So, they my colleagues accepted them but I don’t know that anyone actually recruited or that it made a significant difference to our recruitment to be honest. (R7 – GP recruiter).*

EMIS prompts were used by some of the recruiting sites. These prompts similarly received a mixed reception; whilst they had the potential to keep the study in the minds of clinical staff, they could also become a source of “irritation.”

*Some of them [EMIS prompts] are not always appropriate for the moment and so sometimes they get, they can cause a wee bit of irritation and amongst all our colleagues…(R6 – nurse recruiter).*

*When we were struggling a bit to recruit, he put that [EMIS prompt] in. So [um] yeah, so I think it was on everybody’s mind… (R2 – nurse recruiter*).

Once participants had been recruited to the trial, recruiters found trial procedures straightforward. Most recruiters could complete the recruitment process in 10 to 20 min; some could fit recruitment into a routine clinical appointment slot (circa 15 min). A one-page, A4 flow chart summarising the recruitment process in a step-by-step fashion was praised as a helpful aide-memoire, which recruiters said they used regularly.

That flow chart…was perfect…that just fed you through the, the process very easily… (R4—nurse recruiter)

### Theme 3 – UTI patients’ experiences of CUTI trial procedures

CUTI trial participants used a 14-day electronic diary to rate their symptoms and record any treatments they were taking for their UTI. Women found the diary intuitive and easy to use. This was also the case for women who would not describe themselves as ‘tech savvy.’ Women typically spent around five minutes a day completing it.

*I think one of the best things about it [electronic diary] is the fact that it tells, it gives you the information from before… that’s what you need, to do it properly, you need to see that you put twenty or you put thirty or you put ten because then you can sit and decide exactly where from there you’re going to go…It would have been very difficult I think without that. (T2).*

*It was very easy because it reminded me every day, so it popped up every day that I had to do it, so yeah that was very and it was all very, laid out very well so it, you know, it wasn’t, it was very easy process. (T13).*

As part of the feasibility testing, a symptom rating scale of 0–50 was developed by the CUTI feasibility trial investigators, an adaptation of an existing Likert scale of 0–6 that has been used in other acute UTI trials [18]. Through questionnaire responses in the electronic diary, most CUTI trial participants (31/35, 88.6%) stated that they had no problems using the scale of 0–50 to rate their symptoms [9]. However, during interviews, women indicated that a smaller scale would have been easier to use.

*I mean it might be almost too fine a scale maybe… But, but yeah, people might like that flexibility…(non-trial participant (NT)12)*

*I think I would struggle a bit to determine in which scale I am…because there are several options… (NT10)*

Women’s preferences regarding the diary format were elicited (paper, electronic or a mobile phone application). The electronic diary was the expressed preference of most women, including many older women. Whilst a mobile phone application was typically the expressed preference of younger women, other women, usually older, found using mobile phone applications unfamiliar territory and consequently “daunting.” The electronic diary was seen as more convenient than a paper diary, which was perceived by many to be inconvenient, harder to remember to complete, and with an added hassle of having to post the completed diary back to the research team. The electronic diary was also a popular option with working-age women, who often reported using a computer for their work and consequently checking their emails several times a day.

## Discussion

### Summary

In deciding whether to participate in or recruit to the trial, it was essential to UTI patients and recruiters that the research question was deemed important. For UTI patients, helping others, improving treatments and contributing to research came into play, as well as perceived personal benefits (e.g. avoiding antibiotics). Recruiters needed to perceive that the research was feasible. Using targeted prompts to clinicians in the minor illness clinic/duty doctor clinic helped channel patients from their usual clinical pathway to the research clinics. Using a script to positively frame the delayed antibiotic group helped to overcome patient expectations to receive immediate antibiotics. Using an electronic symptom diary was generally perceived as acceptable by UTI patients, however, the rating scale of 0–50 was perceived as too wide. Incorporating placebo into a future trial was controversial for some UTI patients.

### Strengths and Limitations

Thirty-four interviews were conducted with trial participants, potential participants, and trial recruiters. Incorporating qualitative elements into trials is increasingly recognised as a useful activity, however, the views sought are usually of patients rather than recruiters. We captured recruiters’ views from each of the CUTI trial recruiting sites, including a mixture of nurse and GP recruiters. UTI patients interviewed were of a range of ages and backgrounds. However, there was limited ethnic diversity in our sample despite recruiting non-trial participants from a more ethnically diverse practice. Although our data indicated saturation, with new interview data not meaningfully changing analysis, we acknowledge that insights from patients with more diverse ethnic background may have further developed the analysis. However, we were not able to identify and recruit such patients. The views sought may therefore not be representative of minority ethnic UTI patients.

Triangulation of insights from UTI patients, CUTI trial recruiters, and quantitative findings [9], allowed multiple perspectives on the same phenomena to be explored. For example, through quantitative means, we were able to discern a marked increase in recruitment to the trial after the introduction of strategies including desk prompts, EMIS prompts and a script. However, it is through qualitative means, as outlined above in the present research, that we were able to understand which of these strategies was most likely to have meaningfully contributed to the substantial increase in recruitment. Triangulation also shed light on divergent findings: in the CUTI feasibility trial electronic questionnaires, most women stated that they had no problems using a scale of 0–50. However, interview insights suggest that women would find a smaller scale more user-friendly. This highlights the usefulness of triangulation, which can deepen understanding of particular phenomena [19].

OAG conducted all of the interviews and was also the trial’s CI. This may have predisposed participants to give a more favourable account of their experiences. The CI tried to negate any such potential effect by positioning interviewees as the experts from the interview outset, explaining that the CI was learning from participants (not the other way round), and making it clear that the purpose of the interview was not to gather positive feedback, but rather to collect information to help shape the design of a future trial. We did not receive ethical approval to interview trial decliners, which may have provided additional insights into reasons for not taking part in research. All interviews were conducted in Oxfordshire, which may limit the transferability of the insights gained to other geographical regions.

### Comparison with existing literature

Factors influencing the decision to participate in research trials have previously been evaluated [20–25]. McCann et al*.* use the term ‘conditional altruism;’ people are interested in taking part in trials because they want to help others and additionally perceive potential personal benefits, but disengage if there are too many negatives associated with taking part [20]. A similar theme emerged in the present interview study; women wished to participate in the CUTI trial to help progress research, and additionally perceived potential personal benefits (e.g. access to a novel treatment – cranberry capsules, and potential to avoid antibiotics). The incorporation of a delayed antibiotic trial arm was generally considered acceptable unless the balance tipped too far towards potential harm (e.g. for some, receiving an immediate placebo).

In an overview of psychosocial barriers and facilitators to taking part in research that included 26 systematic reviews, the authors identified that fear was a prominent barrier to taking part in research [26]. Although UTI patients in the present research spoke of fear of the unknown with respect to the interview study, fear was not typically mentioned in relation to participating in the CUTI feasibility trial. This may be due to the perceived low risk nature of taking cranberry capsules and of acute UTIs, compared with other potential interventions and conditions. Distrust in research was also identified in the overview as a barrier to participation in research, and was more common in minority ethnic groups [26]. This may not have been highlighted in the present research given the limited ethnic diversity in our sample.

Placebo-controlled trials have historically been perceived as the ‘gold standard’ in clinical trial design, but can be problematic [27]. Patient reservations about taking placebos can reduce recruitment and retention of participants to clinical trials [27]. In the present interview study, the possibility of being randomly assigned to receive an immediate placebo was off-putting to some women.

Research suggests that high practice workload combined with lack of time are reasons primary care recruiters may struggle to recruit to trials of incident conditions [5, 6]. The importance of keeping trial procedures simple and fast, ensuring that the research topic is relevant to recruiters, is in keeping with the findings of existing studies [7]. Generic prompts, such as ‘pop-ups’ have the potential to overwhelm clinical colleagues and can cause ‘pop-up fatigue’ [28], primary care clinicians may spend around 50 min each day processing such alerts [29].

### Implications for research

The main ‘bottleneck’ to opportunistic recruitment encountered by recruiters was filtering patients from the usual clinical pathway to the research clinics. This interview study suggests that sending targeted prompts to clinicians based in the clinics in which the incident condition typically presents (e.g. minor illness clinic) may be more effective than generic prompts (e.g. desk prompts/electronic pop-ups). SIVs can be used to tailor the approach to the recruiting site in question, according to their existing clinic structures, optimising recruitment processes. Manually typing reminders at the top of electronic lists may be considered labour intensive; ways to automate this process should be explored in future trials. The time between the SIV and sites opening to recruitment should be kept as short as possible. These findings apply to trials of incident conditions in primary care, not just acute UTIs.

The second stumbling block encountered by recruiters was overcoming some women’s expectation of receiving immediate antibiotics. Incorporating a script to help explain groups that deviate furthest from usual practice, or that participants may perceive as less desirable, may facilitate patient participation in trials of incident conditions.

Electronic systems, both for recruiters and trial participants of all ages, are considered acceptable and often preferable to paper-based systems. However, future researchers asking women to rate symptoms should be mindful that a scale smaller than 0–50 may be easier to use.

In order to determine the efficacy of cranberry extract for acute UTI management, it should ideally be compared with a placebo. The incorporation of placebo into trials of acute UTI should be done sensitively, with a careful explanation of the rationale. If women are assigned to receive an immediate placebo, they should additionally receive back-up antibiotics. Working with PPI contributors will be important to help overcome barriers to recruitment.

A future trial of cranberry extract for acute UTI management should include an embedded qualitative evaluation to capture women’s experiences of taking part in a trial of acute UTI that incorporates placebo. Ethical approval permitting, capturing the views of women declining to take part in the trial would provide additional, useful insights. Both a future trial and future qualitative evaluation study should ideally incorporate the views of people from diverse ethnicities. Ensuring ethnic diversity among PPI contributors, and engaging community bridging researchers who speak different languages [30], may improve diversity in recruitment. In addition, using alternative language to describe the interview process, such as a ‘discussion,’ may make the process seem less daunting to prospective participants.

### Implications for clinical practice

The learning from trial recruiters and UTI patients has provided critical insights that will help to shape trial procedures used in an adequately powered trial of cranberry, increasing the chance that such a trial will recruit to target and retain participants. This will, in turn, increase the possibility of an adequately powered trial providing definitive answers to patients and clinicians alike about the safety and effectiveness of cranberry extract in the context of an acute UTI.

## Conclusion

Opportunistic recruitment to trials in primary care is challenging. Learning from patients and recruiters to these trials through qualitative methods can improve recruitment trials and, therefore, research quality. Ensuring that the trial topic was relevant and that trial procedures were straightforward encouraged UTI patients and recruiters alike to engage with the trial. Targeted prompts directed at patient lists in which incident patients are found were perceived to be effective. Using a simple script to help explain a trial group that may be considered less desirable to patients may also facilitate recruitment.

## Supplementary Information

**Additional file 1.** 

**Additional file 2.** 

**Additional file 3.** 

**Additional file 4.** 

**Additional file 5.** 

## Data Availability

The datasets used and/or analysed during the current study are available from the corresponding author on reasonable request.

## Abbreviations

CI: Chief Investigator
CUTI: Cranberry for Urinary Tract Infection
EMIS: Egton Medical Information Systems
PIL: Participant Information Leaflet
RCT: Randomised clinical trial
REDCap: Research Electronic Data Capture
Sentry: Secure entry site for Nuffield Department of Primary Care Health Sciences, University of Oxford
SIV: Site Initiation Visit
UK: United Kingdom
UTI: Urinary Tract Infection
