# Supplementary material for: Enhancing opportunistic recruitment and retention in primary care trials: lessons learned from a qualitative study embedded in the Cranberry for Urinary Tract Infection (CUTI) feasibility trial
Source: BMC Prim Care. 2022 Jul 26;23:184. doi: 10.1186/s12875-022-01796-7 (PMC9315325; doi:10.1186/s12875-022-01796-7)
Supplement: Supplementary file 1 — Additional file 1. [file 12875_2022_1796_MOESM1_ESM.docx]

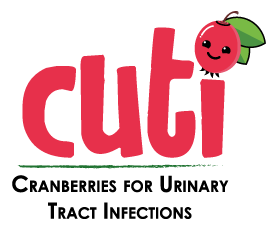


**Recruiter Interview Guide**

**Research Study:** Does cranberry extract reduce antibiotic use for symptoms of acute uncomplicated urinary tract infections? A feasibility study (CUTI): recruiter interview sub-study

**Investigators:** Dr Kome Gbinigie, Dr Anne-Marie Boylan, Professor Michael Moore, Professor Alistair Hay, Professor Carl Heneghan ,Professor Chris Butler.

**Topics to be explored:** Below is a list of questions to be explored in this study. This is intended to be used as a guide and not as a prescriptive tool. As is good practice in qualitative research, the participants’ experiences will be prioritised. This means the same questions may not be asked of all participants in the same order. As new ideas emerge from data collection, new topics and questions may be developed.

**Briefing:**

1) Thank participant for agreeing to take part and introduce self.

2) This interview is for the Cranberries for UTI (CUTI) study. The participant information leaflet has hopefully given you some background about why I’m speaking to you today. I’m going to speak to you about your experience of recruiting to and your thoughts on the CUTI trial, in which we looked at using cranberries to help treat UTIs. I’ll be asking you lots of questions, but you’re the expert in this and I’ll be guided by you. I hope you don’t mind; I might jot down some notes as I go along to help remind me of some important points and things to ask you.

3) If at any time during the interview you do not wish to answer a question, that’s fine. Just let me know.

4) As you know from the information you were given about the study, I would like to digitally record our conversation. The recording will be typed out, but everything you say will be anonymous. Your name and any names you mention, and any places you mention will be taken out, so that if someone read your interview they would not know who you are.

[
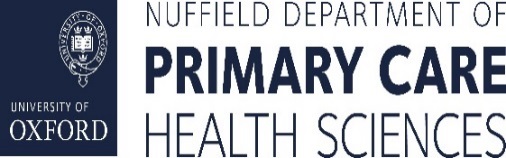
](https://www.google.co.uk/url?sa=i&source=images&cd=&ved=2ahUKEwi0mumojMzcAhVR1xoKHRGQD2IQjRx6BAgBEAU&url=https://www.phc.ox.ac.uk/intranet/communications-engagement/comms/brandguidelines&psig=AOvVaw25IKRdoU5fZKRI-OJ-Rdxv&ust=1533221189259821) [
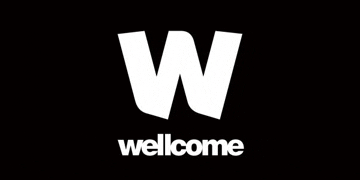
](https://www.google.co.uk/url?sa=i&source=images&cd=&ved=2ahUKEwiuxNPikczcAhWwyYUKHaUYAkAQjRx6BAgBEAU&url=https://jobs.newscientist.com/en-gb/employer/10006940/wellcome-trust/&psig=AOvVaw0mDDZCSW5l1hKxQkGqkRAC&ust=1533222668102409) [
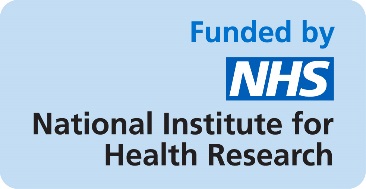
](http://ghrgst.nihr.ac.uk/about-us/)

5) Your interview will remain confidential, unless (as outlined in the information leaflet) it is possible that you or someone else is at risk, but this will be discussed with you first.

6) If at any stage you wish to stop the audio recorder, please let me know.

7) Do you have any questions?

PART A – Before starting CUTI trial recruitment

**1) Could you tell me your initial thoughts on the CUTI trial, before you started recruiting to the study?**

Prompts: How did you first hear about the trial? Did you think it sounded interesting? Did you feel it sounded useful? Did you feel that the study seemed achievable? Did you think it would be easy/hard to recruit to? What did you think about treating a UTI with cranberry? How did you think participants might react to the trial? What made you agree to recruit to this trial?

**2) Before the trial started, a site initiation visit took place with the Chief Investigator. Could you tell me your thoughts on/experience of this?**

Prompts: What did and didn’t go well? Was too much/too little information given? What are your thoughts on the powerpoint presentation? Was the presentation clear? How did the recruitment process seem to you from what was presented? What are your thoughts on the way that electronic recruitment using REDCap/Sentry were explained? Had you used REDCap/Sentry before?

PART B – Recruiting to the CUTI trial

I would now like to ask you a bit about your experience of recruiting to the CUTI trial.

**1) How did you feel about approaching participants to take part in the trial?**

Prompts: What made it easy or difficult? Is there anything that could have improved this?

**2) How did patients generally react when approached to take part in the trial?**

Prompts: How do you think they felt about it? What kind of things did they say? Was it easy/hard to recruit patients? What were the reasons that patients gave for declining to take part? Did you notice if there were any types/groups of patients that were more or less likely to take part?

**3) How did you find the actual process of recruiting participants?**

Prompts: How did you find using REDCap and Sentry systems? Did you encounter any technical problems? Did you need to contact the trial team for help? If so, what was your experience like? How did you find the site file? What do you think about the time that it took to recruit to the trial? Was there any difficulty explaining the different treatment groups to participants and/or issuing cranberry capsules? Did you have any concerns about patients being randomised to the delayed antibiotics group? What was your experience of the scripts for group 3 and the desk prompts?

**4) At the end of the study, you may have conducted an electronic notes review. If applicable, could you tell me your experience of this?**

Prompts: How did you find accessing the electronic notes review form? Was it easy/hard to complete it? Do you think that there are things that we could make easier?

PART C – Post CUTI trial recruitment

I would now like to ask you some questions about your general thoughts on the CUTI trial.

**1) What do you feel could be done to improve the experience for participants (i.e. patient participants) of the trial?**

Prompts: Do you think that there are things that we could make easier? Do you think that there are things that we should/should not do?

2) **2) At the two week follow-up call, participants who have given consent are given information about potentially being interviewed about their experience of being in the study.**

**Can you think of any reasons that people might not like to be interviewed as part of the study?**

**3) What do you feel could be done to improve the experience for recruiters to the trial?**

Prompts: Could we do anything to make it easier? Do you think that there are things that we should/should not do?

**4) We hope to conduct a larger scale trial at a later date. How would you feel about recruiting to this larger study?**

Prompts: Would you require any incentives? Do you think that it’s important to address the questions being asked by the CUTI trial?

**5) Is there anything else you would like to mention that we haven’t talked about?**

This is the end of the interview, thank you very much for taking part.

**END**
