# Supplementary material for: Enhancing opportunistic recruitment and retention in primary care trials: lessons learned from a qualitative study embedded in the Cranberry for Urinary Tract Infection (CUTI) feasibility trial
Source: BMC Prim Care. 2022 Jul 26;23:184. doi: 10.1186/s12875-022-01796-7 (PMC9315325; doi:10.1186/s12875-022-01796-7)
Supplement: Supplementary file 2 — Additional file 2. [file 12875_2022_1796_MOESM2_ESM.docx]

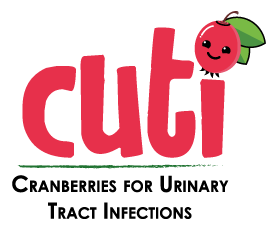


**Patient Interview Guide**

**Research Study:** Cranberries for Urinary Tract Infections (CUTI): Interview Study

**Investigators:** Dr Kome Gbinigie, Dr Anne-Marie Boylan, Professor Carl Heneghan, Professor Chris Butler

**Topics to be explored:** Below is a list of questions to be explored in this study. This is intended to be used as a guide and not as a prescriptive tool. As is good practice in qualitative research, the participants’ experiences will be prioritised. This means the same questions may not be asked of all participants in the same order. As new ideas emerge from data collection, new topics and questions may be developed.

**Briefing:**

**CONTACT A-MB**

1) Thank participant for agreeing to take part and introduce self.

2) This interview is for the Cranberries for UTI (CUTI) study. The patient information leaflet has hopefully given you some background about why I’m speaking to you today. I’m going to speak to you about your experience of having a UTI and your thoughts on the CUTI trial, in which we are looking at using cranberries to help treat UTIs. I’ll be asking you lots of questions, but you’re the expert in this and I’ll be guided by you. I hope you don’t mind; I might jot down some notes as I go along to help remind me of some important points and things to ask you.

3) If at any time during the interview you do not wish to answer a question, that’s fine. Just let me know.

4) As you know from the information you were given about the study, I would like to digitally record our conversation. The recording will be typed out, but everything you say will be anonymous. Your name and any names you mention, and any places you mention will be taken out, so that if someone read your interview they would not know who you are.

[
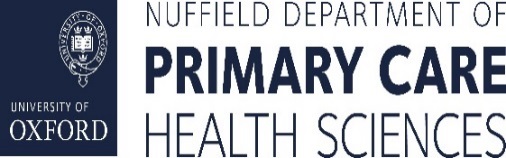
](https://www.google.co.uk/url?sa=i&source=images&cd=&ved=2ahUKEwi0mumojMzcAhVR1xoKHRGQD2IQjRx6BAgBEAU&url=https://www.phc.ox.ac.uk/intranet/communications-engagement/comms/brandguidelines&psig=AOvVaw25IKRdoU5fZKRI-OJ-Rdxv&ust=1533221189259821) [
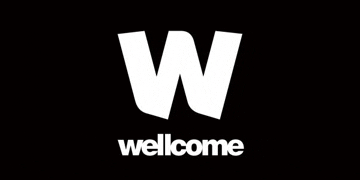
](https://www.google.co.uk/url?sa=i&source=images&cd=&ved=2ahUKEwiuxNPikczcAhWwyYUKHaUYAkAQjRx6BAgBEAU&url=https://jobs.newscientist.com/en-gb/employer/10006940/wellcome-trust/&psig=AOvVaw0mDDZCSW5l1hKxQkGqkRAC&ust=1533222668102409) [
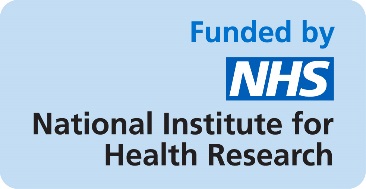
](http://ghrgst.nihr.ac.uk/about-us/)

5) Your interview will remain confidential, unless (as outlined in the information leaflet) it is possible that you or someone else is at risk, but this will be discussed with you first.

6) If at any stage you wish to stop the audio recorder, please let me know.

7) Do you have any questions?

8) Just before I start recording, do you mind if I ask you a few questions about your background? This is just so that we can try to talk to people from a range of backgrounds.

**Age in years**

18-30

31-50

51-64

65+

**Marital status** (Mark with an ‘x’)**:**

Single, never married

Married or domestic partnership

Widowed

Divorced

Separated

**Employment status** (Mark with an ‘x’)**:**

Employed for wages

Self-employed

Out of work and looking for work

Out of work but not currently looking for work (Please see overleaf)

Homemaker

Student

Retired

Unable to work

**What is the highest level of school or degree you have completed** (Mark with an ‘x’)?

v

No schooling completed

Some secondary school

v

v

GCSE level

A-level

v

v

College qualification or equivalent

Trade/technical/vocational training

v

University bachelor’s degree

v

Master’s degree

v

Doctorate degree

v

Other (Please specify)

**Please specify your ethnic** **group** (Mark with an ‘x’)**:**

White

English / Welsh / Scottish / Northern Irish / British

Irish

Gypsy or Irish Traveller

Any other White background, please describe

Mixed / Multiple ethnic groups

White and Black Caribbean

White and Black African

White and Asian

Any other Mixed / Multiple ethnic background, please describe

Asian / Asian British

Indian

Pakistani

Bangladeshi

Chinese

Any other Asian background, please describe

Black / African / Caribbean / Black British

African

Caribbean

Any other Black / African / Caribbean background, please describe

Other ethnic group

Arab

Any other ethnic group, please describe

**GP practice:** _____________________________________________________

**For CUTI Trial participants**

PART A – Before CUTI trial recruitment

**1) I’d like you to tell me all about the last UTI you had. What it felt like, what you thought was wrong, what you did and when you went to see someone about it? Everything about it. So, could you start by telling me what happened from the moment you noticed something was wrong?**

Prompts: What was it like? How did you first realise that you were unwell? What sort of symptoms did you have? What do you think caused it?

**2) What did you do when you realised that something was wrong?**

Prompts: Did you try anything for your symptoms before you contacted your GP/healthcare provider? If so, what did you take? Where did you get it from? Did you ask anyone else for advice? Did you try any over the counter medicines? Did you try any alternative treatments/homeopathy?

**3) What made** **you contact your GP/healthcare provider?**

Prompts: How long after your symptoms started did you contact your GP/healthcare provider? Would you normally have gone in to see your healthcare provider/GP, i.e. if you hadn’t agreed to take part in the CUTI trial? What were you hoping for or expecting from your GP/healthcare provider? Did you find it difficult deciding when to go/contact them? Why is that? Was there any particular trigger that made you contact your GP/healthcare provider?

PART B – Self-care and alternative treatments for UTIs (e.g. cranberry capsules/extract)

I would now like to ask you a bit about your general thoughts on/experience of managing UTIs, self-help and alternative treatments for UTIs.

**1) How do you feel about being prescribed antibiotics for UTIs?**

Prompts: What are your thoughts on and experiences of taking antibiotics? Do you feel that antibiotics are always required? Have you had any problems with them (if applicable)? Do they work for you (if applicable)? What are your thoughts on the cost of getting a prescription for an antibiotic? Does this influence whether or not you contact your GP?

**2) What do you think about treatments that you can buy over the counter?**

Prompts: Have you tried anything other than antibiotics? Do you ever try painkillers? Have you tried/do you know of/what are your thoughts on alternative treatments? Do you have/would you have any concerns about trying these types of treatments? Would you consider them alongside or instead of antibiotics? Where do you go to get non-antibiotic treatments? Do you go to health shops/pharmacy/herbal medicine shops? What are your thoughts on the cost of over the counter treatments? What are your thoughts on the use of cranberry?

**3) Have you ever discussed over the counter/non-antibiotic treatments for UTI with your GP/pharmacist/healthcare provider?**

Prompts: If yes, what did they say? Were they positive, negative or neutral about them? Does your GP/pharmacist/healthcare provider’s thoughts influence how you feel about alternative treatments?

**4)** **What do you do to look after yourself when you have a UTI?**

Prompts: Have you ever tried drinking more fluids/water? Do you think that there are any actions that you can take to prevent/treat UTIs?

PART C – CUTI trial recruitment and experience

I would now like to ask you some questions about your experience of being in the CUTI trial.

**1) How did you find out about the CUTI trial?**

Prompts: Who told you about it?

**2) Why did you take part in the trial?**

Prompts: What were your thoughts when you were told about the trial?

**3) Could you talk me through what happened when you were told about the trial, before deciding to take part?**

Prompts: What was the consultation like? What did your GP/healthcare provider/researcher say?

**4) What happened during the rest of your consultation with the GP/healthcare provider/researcher?**

Prompts: How was the consent process? Which group were you placed into (e.g. antibiotics alone, antibiotics and cranberry capsules, or cranberry capsules and delayed antibiotics? Were the instructions given to you by the person who consented you clear?

**5) Could you tell me what it has been like for you to take part in the CUTI trial?**

Prompts: Were there any problems? Was there anything that might put people off taking part? What were the worst things about taking part? How did you feel about the group that you were put in? Is there a group that you were hoping to be in? Is there a group that you didn’t want to be in? How did you feel about being randomly assigned to a group? What were the best things about taking part?

**6) How did you feel about being asked to provide a urine sample?**

**7) How did you find using the electronic symptom diary?** (Can show paper copy)

Prompts: Were there any difficulties? Was it easy to remember to use the diary? What are your thoughts on completing an electronic diary? How did you find rating your symptoms on a scale from 0-50? Did you complete it using a computer/laptop or a mobile phone? What would be your thoughts on using a mobile phone app? Do you have any thoughts on what the app should look like? What might make it easy for you to use?

**8) You should have received daily email reminders for 2 weeks after you were recruited to the trial to remind you to complete the electronic symptom diary – how was that?**

Prompt: If received, was this acceptable to you? If not received, would this have been helpful/acceptable to you? What would your thoughts be on receiving text message reminders instead?

**9) A member of the research team should have contacted you 2 weeks after you were recruited to the trial – did this happen? If yes, how was that?**

Prompts: Was the length of the phone call okay? Were the questions okay? Did you receive a phone call at day 3? If so, how did you find that?

How did you find being asked to be interviewed? Can you think of reasons that people might not like to be interviewed as part of the study?

**10) Did you have to see a healthcare professional/GP/out of hours/ED/hospital after you were recruited to this study, due to a complication/problem related to this study?** **If yes, could you tell me what happened?**

Prompts: Who did you see? How did you feel? Did you have to take time off work/your usual activities?

**11) Is there anything we could change to make it easier for people to take part in this study?**

Prompts: Do you think that there are things that we could make easier? Do you think that there are things that we should/should not do?

**12) How would you have felt if you could have been randomised to a group that had a placebo (i.e. sugar pill)? Advice to take a placebo, but with a back-up prescription for antibiotics if needed? You would still be able to take painkillers and other OTC medicines as needed.**

Prompts: How would you feel if you were put in this group only if you were happy to try to delay antibiotics? What would your thoughts be on a study in which you receive immediate antibiotics (with PLA or cranberry) if you don’t want to delay antibiotics, and in which you receive immediate cranberry or PLA (with back-up antibiotics) if you are willing to delay taking antibiotics? Is there any group that you would not like to be in? Why?

**13) How would you feel if we focussed the study only on women with milder symptoms? In this case, we might have two groups to see whether we can help them to delay antibiotics. Either back-up antibiotics, or cranberry+ back-up antibiotics.**

Prompts: How would you feel about this design? Would you take part? Do you think that this answers a helpful question?

**14) Is there anything else you would like to mention that we haven’t talked about?**

That is the end of the interview, thank you very much for taking part.

**END**

**Contact A-MB**

**For non-CUTI Trial participants**

PART A – Your UTI experience

**1) I’d like you to tell me all about the last UTI you had. What it felt like, what you thought was wrong, what you did and when (or if) you went to see someone about it? Everything about it. So, could you start by telling me what happened from the moment you noticed something was wrong?**

Prompts: Prompts: What was it like? How did you first realise that you were unwell? How did you know it was a UTI? What sort of symptoms did you have? How long did it take for you to feel completely better?

**2) What did you do when you realised that something was wrong?**

Prompts: Did you try anything over the counter for your symptoms (before you contacted your GP/healthcare provider)? Did you ask anyone else for advice?

**3) What made** **you contact your GP/healthcare provider (if applicable)?**

Prompts: If yes, was there any particular trigger that made you contact your GP/healthcare provider? How long after your symptoms started did you contact them? What were you hoping for or expecting from your GP/healthcare provider?

**4) (If applicable) Did you have any examinations or tests?**

Prompts: What sort? How did these go? If none were done, were you expecting any?

**5) (If applicable) What did the GP/healthcare provider suggest to improve your symptoms?**

Prompts: What advice were you given? Were you advised to use any particular medications?

**6) (If applicable) Did your doctor prescribe you antibiotics?**

Prompts: How did you feel about this? Did you feel you needed them?

**7)** **(If applicable) How do you feel about the consultation that you had with your GP/healthcare provider?**

Did you feel you got the right treatment? Did you feel you got the right information? **8) What happened during the rest of your UTI episode?**

How long did it take for your UTI to get better? Did you have any problems with the treatments that you tried? (If applicable) Did you have to return to see your GP/ a healthcare professional? If so, why?

PART B – Self-care and alternative treatments for UTIs (e.g. cranberry capsules/extract)

I would now like to ask you a bit about your thoughts on/experience of managing UTIs, self-help and alternative treatments for UTIs.

**1) How do you feel about being prescribed antibiotics for UTIs?**

Prompts: What are your thoughts on and experiences of taking antibiotics? Do you feel that antibiotics are always required? What are your thoughts on the cost of getting a prescription for an antibiotic? Does this influence whether or not you contact your GP?

**2) What do you think about treatments that you can buy over the counter?**

Prompts: Have you tried anything other than antibiotics? Do you ever try painkillers? Have you tried/do you know of/what are your thoughts on alternative treatments? Do you have/would you have any concerns about trying these types of treatments? Would you consider them alongside or instead of antibiotics? Where do you go to get non-antibiotic treatments? Do you go to health shops/pharmacy/herbal medicine shops? What are your thoughts on the cost of over the counter treatments? What are your thoughts on the use of cranberry?

**3) Have you ever discussed over the counter/non-antibiotic treatments for UTI with your GP/pharmacist/healthcare provider?**

Prompts: If yes, what did they say? Were they positive, negative or neutral about them? How might their thoughts influence how you feel about non-antibiotic treatments for UTIs?

**4)** **What do you do to look after yourself when you have a UTI?**

Prompts: Have you ever tried drinking more fluids/water? Is there anything you feel you can do to prevent you from getting a UTI?

PART C – Your thoughts on the CUTI trial

You will be aware that at the moment we are asking women who have a UTI to take part in a trial (called the ‘CUTI’ trial) in which they are being randomly assigned to different treatment groups to test whether cranberry capsules can help treat urinary tract infections (UTIs). *The main aim of this trial is to test whether the way we do this research works and is acceptable to the women who take part (called a ‘feasibility trial’).* This will help us to plan a similar but bigger trial.

I would like to tell you a bit about the trial, and then find out how you might feel if you were asked to take part.

Women with a UTI who agree to take part in the study are randomly divided randomly into three different groups:

1) Advice to take antibiotics

2) Advice to take antibiotics and cranberry capsules

3) Advice to take cranberry capsules alone. Women in this group would be given a delayed prescription for antibiotics, with advice to start taking antibiotics if their symptoms don’t get better in the next 3-5 days.

- Women will be advised to seek medical help if they develop any symptoms of an upper UTI (e.g. vomiting, fever, back pain)

**1) What are your thoughts on this?**

How does this trial sound to you? Is it something you might be interested in taking part in? How important/valuable do you think this research is? How might you feel about being randomly being put into a group? Is there any group that you might like to be put in? Is there any group that you might not wish to be put in? Is there anything that we could do that you feel might make people feel happier to be in group 3 (cranberry+ delayed abx)?

- Women taking part are emailed a link to an electronic symptom diary to complete over a period of up to 2 weeks (until all symptoms have resolved), with daily reminders to complete the diary. The main part of the diary is rating symptoms daily on a scale of 0-50 (Show them the paper version of the scale)

**2) What are your thoughts on this?**

Prompts: Does it sound difficult? Would you remember to complete the symptom diary daily? How might you find completing an electronic diary? What would your thoughts be on receiving text message reminders instead? What would you think about having the diary as a mobile phone app? Do you have any thoughts on what the app should look like? What might make it easy for you to use?

**3) What are your thoughts on the scale?**

Prompts: Do you think it’s clear? How do you think this compares to a smaller scale (e.g. 0-6)?

- Participants should also receive a call from someone in the research team 2 weeks after they start the trial as a final encouragement to participants to complete the symptom diary? Participants are also asked whether they are happy to receive information about being interviewed as part of the study.

**5) What are your thoughts on this?**

Prompts: How would you find this? What might be the positives and negatives in your opinion? Can you think of reasons that people might not like to be interviewed as part of the study?

**6) What are your thoughts overall on the trial that I’ve described to you?**

Prompts: Do you think that you would take part? Why, or why not? Do you think that there is anything that could/should be changed/adjusted/removed/added in? Do you think that there is anything that might put people off taking part? What do you think might encourage people to take part?

**7) How would you have felt if there were an additional group that you could be randomised to, in which you would take a placebo (i.e. sugar pill)? Advice to take a placebo, but with a back-up prescription for antibiotics if needed? You would still be able to take painkillers and other OTC medicines as needed.**

Prompts: How would you feel if you were put in this group only if you were happy to try to delay antibiotics? What would your thoughts be on a study in which you receive immediate antibiotics (with PLA or cranberry) if you don’t want to delay antibiotics, and in which you receive immediate cranberry or PLA (with back-up antibiotics) if you are willing to delay taking antibiotics? Is there any group that you would not like to be in? Why?

8) **13) How would you feel if we focussed the study only on women with milder symptoms? In this case, we might have two groups to see whether we can help them to delay antibiotics. Either back-up antibiotics, or cranberry+ back-up antibiotics.**

Prompts: How would you feel about this design? Would you take part? Do you think that this answers a helpful question?

**9) Do you have any more questions about the CUTI trial?**

**10) Is there anything else you would like to mention that we haven’t talked about?**

That is the end of the interview, thank you very much for taking part.

**END**

**Contact A-MB**
