# Supplementary material for: Enhancing opportunistic recruitment and retention in primary care trials: lessons learned from a qualitative study embedded in the Cranberry for Urinary Tract Infection (CUTI) feasibility trial
Source: BMC Prim Care. 2022 Jul 26;23:184. doi: 10.1186/s12875-022-01796-7 (PMC9315325; doi:10.1186/s12875-022-01796-7)
Supplement: Supplementary file 3 — Additional file 3. [file 12875_2022_1796_MOESM3_ESM.docx]

**Additional file 3 - Script for recruiters**

"If you are randomly placed in the third group, you have the chance to access a non-antibiotic treatment. You will still receive a back-up prescription for antibiotics, so you are in control and can take the antibiotics whenever you feel you need them. We do ask that you try to delay taking the antibiotics, as you might get better with the cranberry alone and avoid antibiotics altogether. However, if you feel you need to take the antibiotics at any point, that’s fine."
