# Supplementary material for: Enhancing opportunistic recruitment and retention in primary care trials: lessons learned from a qualitative study embedded in the Cranberry for Urinary Tract Infection (CUTI) feasibility trial
Source: BMC Prim Care. 2022 Jul 26;23:184. doi: 10.1186/s12875-022-01796-7 (PMC9315325; doi:10.1186/s12875-022-01796-7)
Supplement: Supplementary file 4 — Additional file 4. [file 12875_2022_1796_MOESM4_ESM.docx]

**Additional Table 1** – Key learning points for recruiting to trials of incident conditions in primary care

| **Theme** | **Learning points** | **Supporting quotes** |
| --- | --- | --- |
| Theme 1 | - Patients often take part in research to help advance medical knowledge, facilitated by additional perceived personal benefits - The idea of a research interview may be an unknown entity for patients. Some might find the idea anxiety-provoking. Using alternative language, such as a ‘discussion,’ may allay fears and encourage recruitment. - Recruiters are more likely to take part if they feel the research answers an important question and is achievable - The use of an immediate placebo arm in a UTI study is likely to dissuade some women from taking part. If used, the rationale for including placebo should be carefully explained, and back-up antibiotics should be provided for an immediate placebo arm. | *I suppose some people [who decline to be interviewed] might be worried about what you were going to ask, whether that was probing into their personal, I don’t know, their, their body maybe or, or, you know, whether or not they thought they had to have an examination or answer some more questions.*(T14)  *Maybe not call it an interview, maybe call it a, a check-in or I don’t know, you could call it [pause 8 secs] study completion or information check-up or even just something like a discussion…I think something that’s maybe a bit, something doesn’t instantly make people think about being interviewed.*(T12)  *Fundamentally the question that’s being asked is a sensible question. It’s a very simple study design and simple to fulfil if you like, you know, patient comes in, gets assessed, gets a product, not too many visits, not too onerous on our staff, not too onerous on the patient and potential for great impact.* (R7 – GP recruiter)  *I’m not sure I’d have been as happy because the whole point of going to the doctor was that you want it to get better and if you theoretically aren’t getting any treatment…theoretically you might not be getting better… I think I would have thought twice about it.* (T14)  *It [placebo] would annoy me because I’d just put off the inevitable for nothing for sugar pills…Absolutely not interested.* (T9)  *I really don’t believe that mind of matter could clear up an infection…but I wouldn’t be too worried if there were antibiotics to follow…*(T6) |
| Theme 2 | - Real-time communication/messages targeted at clinicians working in the clinics in which patients with incident conditions usually present (e.g. minor illness clinics) are likely to facilitate the flow of patients from their usual clinical pathway through to research clinics. - Recruiters value a concise (A4) flow chart summarising recruitment processes. - The use of a script to explain trial groups that may be considered less desirable (e.g. delayed antibiotics) can aid recruiters’ interaction with potential participants, and hence also recruitment. - EMIS pop-ups may cause ‘irritation’ to clinicians if they are not deemed appropriate for the moment. If desk prompts are used, they should be large and eye-catching. - Ensuring that trial procedures are quick and straight-forward is likely to help opportunistic recruitment, potentially enabling recruiters to recruit patients in their routine clinical slots. - Recruiters value regular communications from the central trial team. - Ensure that all relevant information about the trial and trial processes is given at the Site initiation Visit (SIV) - The SIV should take place as close as possible to site opening | *When I came in to do research on X’s day when she’s in minor illness, I’d look through her list and I’d be able to spot the urine infections because they’re identified on the, the patient on the patient appointment system online…I would Sophos her, message her instantly and say, “There’s a number of UTIs so I’ll, I can take them.* (R2 – nurse recruiter)  *Yeah, I did use them [the script] actually because I think it’s always useful just to make sure that, to reassure myself that I am saying the right thing as well and that you know, people, people kind of, I don’t know take it with more authority if you’re reading it from the study people maybe.* (R5 - nurse recruiter)  *I’ve only got ten minutes appointments and I was managing to do it [recruit participants] in that…* (R6 - nurse recruiter)  *I thought, you know, we had con-, you know, constant email contact which was really good, so I don’t feel at any point if we’d been struggling or anything it will, we wouldn’t have been able to get hold of you, so that was really good.* (R2 - nurse recruiter)  *The flowsheet sheet that you gave us, almost like a crib sheet kind of thing. I found that really useful and followed that through.* (R5 - nurse recruiter)  *Site initiation visits; they* *should always be as close to the starting date as possible….* *Because what happens, what often happens then is people forget about the study when it goes into the long grass for a while and then people then need a refresh and so it almost wastes time by going through the refresh period again.* (R8 – GP recruiter) |
| Theme 3 | - Participant completed symptom diaries should be intuitive, and daily entries should not take long to complete (ideally no more than five minutes). - If patients are required to provide daily symptom ratings, it is useful for them to see their ratings on previous days. - Daily reminders to complete the diary are helpful (via email or text message); participants do not find them intrusive. - When designing symptom diaries for studies, consider the age group of the study participants. If the participants are exclusively younger (aged under 50 years), a mobile phone application may be the most appropriate format. If the study will include older people or people of all ages, then an electronic diary may be more appropriate. - Many older people are happy to use an electronic diary and prefer this to using a paper diary. - Patients may find it easier to use scales that are smaller than 0-50. | *It only took a few minutes. It wasn’t arduous at all.* (T5)  *I’m not very technically minded and I’m not good on the computer. I’ve only learnt to text and do emails and things properly over the last few years….My husband…he looked at it first and he said to me, “It’s so straight forward,” and I didn’t have to have any more help...I thought it was good... especially my age group, you do need to do that because people just can’t be bothered to have anything too arduous… (T2)*  *That [email reminder] was quite useful because if I was really busy to have the reminder, it sort of prompted me, ‘Oh, yeah, I’ve got to do that this evening.’* (T10)  *It was good…yeah appreciated the emails every day to remind me to do it…it would have been quite easy to have forgotten so the emails coming in were quite good.* (T14)  *I’m generally on my computer any given point during the day or on my tablet or something like that, it was just easy because I just would just flick into it [electronic diary] and flick out. Whereas, this [paper diary] would perhaps be a bit more of a conscious must remember to pen and paper, you know what I mean ….*  (T13 – 68 years)  *If you wanted my thoughts on how to improve it [the diary], you could have an App…an App would be nice and easy* (T12 – 32 years)  *I’m still learning how to do an App. I think if I went now and they said. “We’re going to do it [the diary] all by App.” I’d go, “Please, no.” I probably wouldn’t want to do it…unless it was unbelievably simple. But if I had cystitis and I was still unfamiliar with doing Apps, it would be a deterrent because I would not be feeling like making the extra effort of being stressed out by the bally machine.* (NT6 – 62 years)  *I mean I, I’m thinking, ‘Fifty, gosh that’s big; that’s huge, you know, am I at death’s door at that point?’* (NT4)  *It would have been easier to have filled in [scale of 0-6] because you didn’t have to think about it quite so much.* (T14) |
